# Inertial Measurement Unit Sensors in Assistive Technologies for Visually Impaired People, a Review

**DOI:** 10.3390/s21144767

**Published:** 2021-07-13

**Authors:** Karla Miriam Reyes Leiva, Milagros Jaén-Vargas, Benito Codina, José Javier Serrano Olmedo

**Affiliations:** 1 Center for Biomedical Technology (CTB), Universidad Politécnica de Madrid, 28223 Madrid, Spain; milagros.jaen@ctb.upm.es (M.J.-V.); josejavier.serrano@ctb.upm.es (J.J.S.O.); 2 Engineering Faculty, Universidad Tecnológica Centroamericana UNITEC, 211001 San Pedro Sula, Honduras; 3 Didactic and Educational Research Department, Universidad de La Laguna, 38204 San Cristóbal de La Laguna, Spain; bcodina@ull.es; 4 Spanish Blind Organization (ONCE), 38003 Santa Cruz de Tenerife, Spain; 5 Networking Center of Biomedical Research for Bioengineering Biomaterials and Nanomedicine, Instituto de Salud Carlos III, 28029 Madrid, Spain

**Keywords:** accelerometer, assistive technologies, gyroscope, IMUs, visually impaired, usability

## Abstract

A diverse array of assistive technologies have been developed to help Visually Impaired People (VIP) face many basic daily autonomy challenges. Inertial measurement unit sensors, on the other hand, have been used for navigation, guidance, and localization but especially for full body motion tracking due to their low cost and miniaturization, which have allowed the estimation of kinematic parameters and biomechanical analysis for different field of applications. The aim of this work was to present a comprehensive approach of assistive technologies for VIP that include inertial sensors as input, producing results on the comprehension of technical characteristics of the inertial sensors, the methodologies applied, and their specific role in each developed system. The results show that there are just a few inertial sensor-based systems. However, these sensors provide essential information when combined with optical sensors and radio signals for navigation and special application fields. The discussion includes new avenues of research, missing elements, and usability analysis, since a limitation evidenced in the selected articles is the lack of user-centered designs. Finally, regarding application fields, it has been highlighted that a gap exists in the literature regarding aids for rehabilitation and biomechanical analysis of VIP. Most of the findings are focused on navigation and obstacle detection, and this should be considered for future applications.

## 1. Introduction

According to the World Health Organization, about 28% of the global population (2.2 billion) is visually impaired or blind [1]. Vision impairments have different limitations, including distance and near-vision impairment. As technology advances, there is a need to develop high-quality assistive systems for the inclusion of visually impaired people (VIP) into a technological world to improve their Quality of Life (QoL) and to facilitate daily challenges such as finding and keeping a job, mobility, using public transport, and doing physical activity (PA) [2,3,4,5,6]. With the ongoing progress in computer science, such as deep learning, and in hardware development, such as sensor miniaturization, researchers have developed human activity recognition (HAR) algorithms that enable automatic feature extractions [7,8,9], for instance, by using inertial sensor’s acquisitions as input data [10,11,12,13,14].

In addition, there are great advances in miniaturized sensors capable of providing parameters of moving objects, such as position and velocity [15,16,17]. The fusion of the advances in both sensors and artificial intelligence has led to many projects that seek to support VIP in navigation [18,19,20,21,22,23,24,25,26,27], traveling [28,29,30,31], representation of the real world [32,33,34,35], obstacle detection on wayfinding [29,36,37,38], assistant robots [39,40], and other applications for general mobility, for monitoring and improving PA, and for sports participation of the VIP [41,42,43,44]. The application spectrum is extensive and may include even sensor fusion for monitoring the vital signs of guide dogs in training [45]. However, a large quantity of these systems are designed to provide VIP with information obtained from their surroundings. Electronic Travel Aid Systems (ETAS) [3] are one of the most studied assistive technologies and, according to the recent state of the art, wearable assistive devices for the visually impaired can be divided into two categories: Video camera-based ETAS and Sensorial network ETAS. Sensorial network ETAS are based primarily on GPS, BLE beacons, RFID, Ultrasound sensors, and Infrared sensors [3,46].

An important factor constraining the development of these assistive technologies is that there are limitations regarding the accuracy of these systems. Another important factor is poor acceptance by the blind community, which is a factor related to the limitation of the visual rehabilitation programs in which these systems should be included [47].

Technologies based on inertial measurement unit sensors (IMU) are used in a large and ever-growing number of applications such as intelligence guidance, mineral exploration, self-driving robots [15], full-body motion tracking [48,49,50,51,52,53], and navigation as well [54,55]. IMUs are widely used because they provide positioning information based on the dead-reckoning method, which determines the current position based on estimates of velocity and heading, departing from a known previous position. This type of navigation and tracking information is useful in areas where infrastructure-less positioning systems are required [56], including VIP applications.

There is a large amount of literature on the use of inertial sensors to estimate position and orientation. However, as mentioned before, sensor acquisition in ETAS and other VIP systems developed for navigation and assistance are not primarily IMU-based technologies, although their sensing includes IMUs data. This is due to integration drift, which is a known disadvantage of IMUs that can generate position errors in the dead-reckoning method. To face the drift error, many authors suggest incorporating inertial measurement as part of the acquired sensing and fusing the values with the sensing of optic sensors and global positioning systems (GPS) also to include the drift reduction algorithms, which greatly contribute to a more accurate positioning within IMU data [56,57,58]. As mentioned before, there is a wide range of applications for IMUs. The aims of this systematic review are as follows: (1) to provide an overview of current state of the art research and development on technology that implements IMU sensors in support of VIP; (2) to provide an understanding of how IMU sensors work and how they are used in current developments; (3) to review challenges currently faced in research focused on assisting VIP; (4) to explore application fields, besides those for navigation, in which these types of sensors can be used to support VIP. To enhance reproducibility, the details of the procedure are provided; this is a thematic review in which we pre-selected for content as described below and in which additional relevant findings are discussed.

## 2. Materials and Methods

### 2.1. Literature Search Method

Literature searches were performed in the IEEE Xplore (IE), Web of Science (WoS), and PubMed databases. Considering rapid advances in technology, we focused on articles published in the last 5 years (until December 2020) to give an overview of the most recent developments. Searches were performed in IE, WoS, and PubMed on 15 December 2020. Only articles written in English were considered. Our screening was filtered in two stages: a general search and a refinement in the three databases. Terms used in the general search were (IMU* OR accelerometer OR gyroscope OR magnetometer OR inertial measurement). For the refinement, the terms were (visually impaired OR blind OR visual impairment).

### 2.2. Eligibility Criteria

The following criteria were used to select the articles included in this review: (1) articles with proposed systems including IMU sensor technology with at least one kind of measurement (accelerometer, gyroscope), (2) in the implementation of IoT systems on the developments and (3) rehabilitation or physical monitoring of daily life activities and (4) in publications within experimental results of their developments, including the participation of VIP or blindfolded (BF) volunteers.

### 2.3. Inclusion Criteria

**Figure 1:**
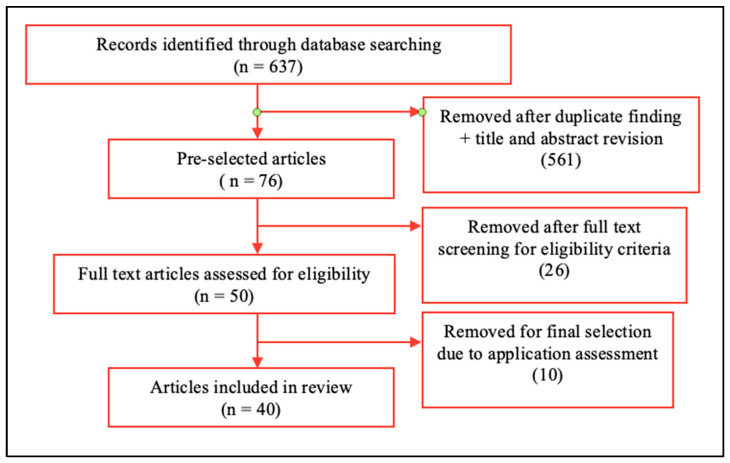
Deep analysis article selection process after database searching.

The initial search resulted in 637 articles (IEEE = 85, WoS = 324, PubMed = 264). To be included in this review, the aim of an article was required to be a development to aid VIP exclusively; articles regarding navigation, human motion, or PA monitoring not developed for VIP were excluded. After applying the selection criteria and removing duplicates, 40 articles remained to be reviewed (Figure 1).

## 3. Results

The articles were summarized and divided into four categories according to the inertial sensor used, “sensor input”. The first section discusses nine articles reporting to use accelerometer input, the second section considers four articles that used gyroscope input, the third and four sections discuss 13 articles using both accelerometer and gyroscope input and 14 articles reported that used accelerometer, gyroscope, and magnetometer input.

Then, the usability, the application trends, and the artificial intelligence incorporation in the reviewed articles are discussed. Note that in the extension of this section, the role of the inertial measurement unit in each development is highlighted; however, most of the selected articles integrate sensor fusion in which other types of sensors (non-inertial) are used.

### 3.1. Accelerometer

As shown in Table 1, 23% of the reviewed articles reported the use of an accelerometer. An accelerometer measures the external specific force acting on the sensor, which consists of both the sensor’s acceleration and the acceleration due to the earth’s gravity. The accelerometer input served several purposes, including position estimation, monitoring of physical movement, and vibration detection. The most frequently used accelerometers included ActiGraph from Actigraph Corp, ADXL from Analog Devices, and KXR from Kionincs.

The Actigraph Corp wearable accelerometers were used in many clinical trials found in the systematic review. The accelerometers were used to measure PA within the VI community, with a major experimental focus on kids and older adults [60,61,62]. Since the blind spend more time in sedentary activities, trials attempted to determine relationships between falls and levels of PA [63]. These wearable sensors present output data of the three-accelerometer axis independently and provide activity counts as a composite vector magnitude of the axis. For instance, familial trials were conducted to correlate PA between VI, their parents, and siblings [66]. While in [65], the authors studied the PA of children with VI during different segments of the school day from the special school for VI in Xingqing Districts in Yinchuan, China. In this trial, a total of 600 min acceleration was recorded per day, and this input was analyzed within the ActiLife Lifestyle Monitoring System from Actigraph.

Nkechinyere et al. [67] developed software to identify specific daily activities performed by VI and elderly persons. The system uses a wearable accelerometer sensor to collect data that is then submitted to neural network regression (NNR) to characterize each activity as standing, sitting, bending, lying down, or walking. Falls and critical falls are also identified. In this work, the velocity–acceleration measurements are converted to gravity–acceleration by multiplying velocity by sensitivity on each axis. Then, gravity acceleration (G) is calculated using the sum of the squares of the *X*, *Y*, and *Z*-axis in order to remove negative values, and a G target value is representative for individual neural network training.

**Figure 2:**
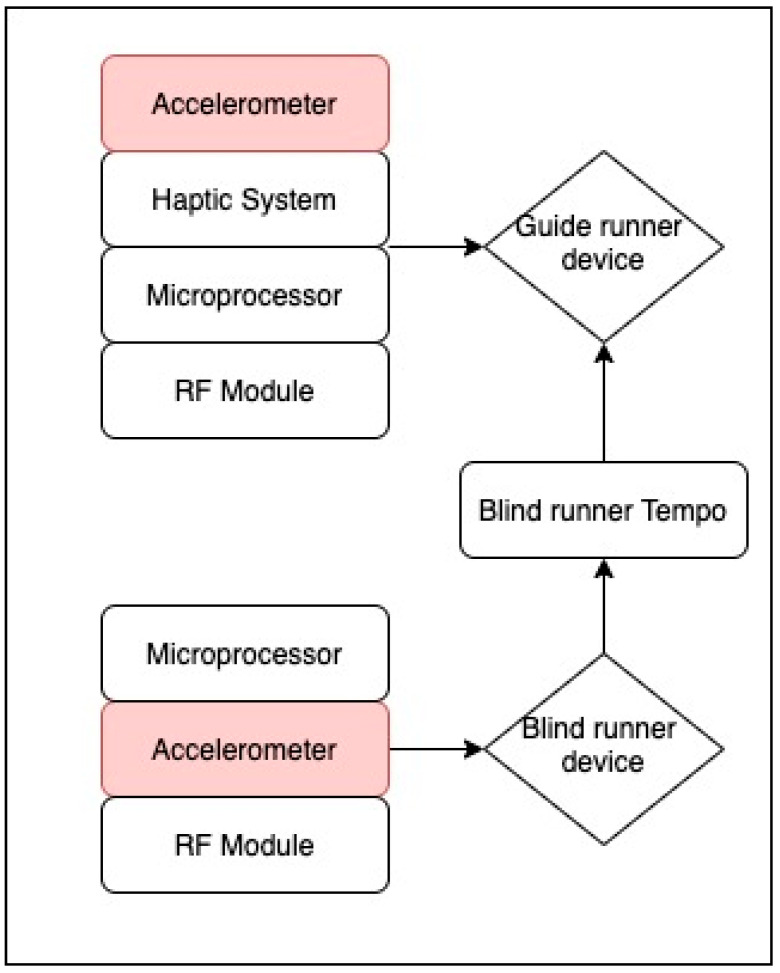
Representation of the algorithm for synchronized running proposed by [64].

Vibration or shocks could also be determined by thresholds fixed for each axis. Case in point, within the system of Hirano et al., the vibration of a KXR94-2050 3-axis accelerometer sensor was used to allow blind runners to synchronize and match their running tempo with sighted guides (see Figure 2). The algorithm in this system was designed so that when the blind runner’s foot touches the floor, a vibration signal was induced in the guide runner’s ankle, allowing for synchronization of the race pace [64]. The algorithm was tasked with identifying foot strikes using a low-pass filter applied to samples. The peak acceleration values caused by foot strikes were detected and sent as vibrotactile feedback through a transducer. An acceleration threshold value (4.74 m/s^2^) was previously settled upon by trial.

The work of [59] presents the design and usage of two assistive technologies for VIP that use an ADXL345 accelerometer: a vibrotactile belt and a stereovision system. The vibrotactile belt (NavBelt modified [68]) was connected to ultrasonic sensors (LV-MaxSonar-EZ0) and to the accelerometer. The acceleration values were used to detect and eradicate unnecessary vibrations when detecting user motion, returning user movement and velocity information. The motion and velocity of a user can be recognized using acceleration by detecting stationary periods with the Zero Velocity Update algorithm [18].

### 3.2. Gyroscope

A gyroscope measures angular velocity: the rate of change of the sensor’s orientation. Thus, the integration of gyroscope measurements provides information about the orientation of the sensor. Four articles reported using gyroscope input for different roles within sensor fusion, as shown in Table 2.

In what can be considered a “rehabilitation” application, the authors of [72] used the gyroscope information to characterize long white cane usage in VI volunteers. In their system, the velocity of the cane’s sweeping movement was obtained by analyzing the gyroscope’s *Z*-axis signal. The sweeping frequency corresponded to the number of complete sweep cycles performed per second. The sweeping speed was defined as the angular velocity during the sweeping period. In addition, the authors experimented with grasping characteristics based on the positions of the thumb and index finger. They added an optoelectronic motion tracking system (QTM/Oqus, Qualisys AB) to obtain accurate cane orientation angles related to tilt, grip rotation, and sweeping movement.

On a different device proposed to help blind people detect stairs [69], the gyroscope output was used to determine if a change in the distance between the user’s head and the ground was due to head tilt or to the user stepping down or up. The method used an ultrasound sensor to measure the distance between the user’s head and the ground. The measured distance was compared to a reference distance that corresponded to the floor. So, to establish the reference distance, the gyroscope measured the α angle (corresponding to the head tilt); therefore, the reference distance was A/(sin α + cos α), where A corresponds to the distance from the head tilt to the floor by trigonometry.

With the gyroscope values, the buzzers also could provide information about the distance from the step as well as the height and depth of the step; in this system, a MPU-6050 IMU was used to obtain the tilt angle. The same sensor was used in [70], where the approach was detecting rotation and movements in an automated smart cane. In this system, a high-frequency sound wave was emitted, and its return was used to calculate the distance to an object. Since the device moves continuously while walking, the bottom sonar sensor was fixed in its initial place to detect high surfaces. The gyroscope values were used to control the servo motor; when the sensor value deviated from the fixed value, the servo rotated and returned to the initial fixed value.

Oommen et al. [71] developed a prototype to aid VI swimmers to train with more independence. The device consisted of a smartphone attached to the waist of the swimmer and Voice Recognition Technology (VRT) as the interface, so the swimmer could manipulate the application communicating to the VRT with waterproof Bluetooth earphones. With the help of the camera facing the bottom of the pool and the gyroscope data from the smartphone, the algorithm alerted the swimmer when the end of the lane was near and when the swimmer drifted sideways. The gyroscope was sampled at a minimum rate of 20 times per second and was used to correct the camera images for swimmer roll during strokes. The inertial measurement was essential to determine the orientation of the device and of the gravity vector in the navigation frame. This meant that camera frames were processed only when the device was parallel to the bottom lines, providing a correct estimation.

### 3.3. Accelerometer and Gyroscope Fusion

Accelerometers and gyroscopes are frequently used together in navigation situations when the position and orientation (i.e., attitude) of a device or person are of interest. Articles that reported using input from both accelerometers and gyroscopes sensors represented 33% of those reviewed. As expected, all dealt with navigation aid applications. The most frequently cited IMU was the MPU-6050 from TDK InvenSense, which has a Digital Motion Processor (DMP) to the fusion of the three-axis accelerometer and three-axis gyroscope; more details are described in Table 3.

Croce et al. [73] designed a system where a smartphone camera was the main sensor, which was used to detect special paths such as colored tapes or a painted line. It was also a tracking system based on the integration of a MPU6500 IMU. The authors used the accelerometer values for “Activity recognition”; the accelerometer covariance along the three axes was analyzed to determine if the user is standing still or walking. For heading estimation (direction of the user), the gyroscope data were used to identify the smartphone reference frame with respect to the navigation frame. To estimate the user position with respect to the navigation frame, the *Z*-axis of acceleration (vertical acceleration) is analyzed to identify steps, while the minimum and maximum vertical acceleration signals are retrieved for peak detection and zero crossings. These features helped to evaluate cardinality so the algorithm could be reliable with different users and different walking speeds. Displacement (s) was evaluated using the algorithm proposed by Weinberg for MEMs in 2002 [74]: $\Delta s=\beta \sqrt[{4}]{\alpha {\left(k\right)}^{M}-\alpha {\left(k\right)}^{m}}$, where $a{\left(k\right)}^{M}$ is the actual time (k) maximum and $\alpha {\left(k\right)}^{m}$ is the actual time (k) minimum of vertical accelerations, and β is the average length of a step. Finally, sensor fusion with the Computer Vision and PDR algorithms was done by implementing an Extended Kalman Filter. In this model, other parameters, such as angular velocity and direction of the user velocity, were provided by the IMU for the discrete time state model.

**Table 3:** Summary reviewed articles in the accelerometer and gyroscope fusion section.

Role	IMU	Sensor Fusion	RE	Ref.
Pedestrian dead reckoning	MPU-6050	RGB-D camera, GPS	0.41 m	[73]
Motion detection	MPU-6050	RGB camera, ultrasonic sensor	-	[75]
Position estimation and orientation	MPU-6050	CMOS camera, line laser	0.4–1 m	[76]
Fall detection and attitude estimation	Not specified	RGB-D camera, GPS, velocity sensor	-	[77]
Fall detection	Smartphone IMU	Ultrasonic sensor, GPS	10–20 m	[78]
Orientation	MPU-6050	GPS, ultrasonic, and wet floor sensors	-	[79]
Attitude estimation	Not specified	RGB-D camera	-	[80]
Step counting	Not specified	Ultrasonic sensor, GPS	-	[81]
Orientation and Height estimation	Not specified	RGB-D camera	-	[82]
Heading estimation	Not specified	GPS, compass	2.9–1.7 m	[83]
Angular velocity and acceleration	Smartphone IMU	Strain gauges	-	[84]
Pose estimation	LSM9DS1	RGB-D camera	-	[85]
Orientation of the head and hand	BMI055 Bosch	UWB FMCW radar sensor	-	[86]

Silva & Wimalaratne [75] used the MPU-6050 inbuilt motion fusion for image deblur processing in an optical obstacle detection belt. This was necessary because the camera’s continuous movement on the body causes image blurring. The deblur process was done by providing the approximate trajectory of the camera motion. In addition, Ref. [78] used it to create fall alarms in a proposed prototype of an intelligent walking stick for VI and elderly people. The hardware sensing was composed of ultrasonic sensors and GPS. The IMU module monitors the posture of the crutches in real time and verifies whether the horizontal angle and the acceleration direction of the crutches are normal so that a fall can be detected.

Chen et al. [77] reported another way to detect falls using inertial measurement sensors. Among other characteristics, fall detection using altitude estimation is based on an algorithm that processes values from inertial sensors in real time. The pitch angle represents the *Y*-axis rotation (body’s backward pitch); the roll, the *X*-axis rotation (body side-slip angle from left to right); and the yaw, the rotation angle around the *X*-axis (rotation angle of the body from left to right). The authors used a Kalman filter algorithm to suppress noise and improve the reliability. Also, they collected additional information (angular velocity and acceleration) to improve the method for user safety.

An assistive device called NavCane [79] was developed to aid VIP finding obstacle-free paths. The NavCane can detect wet floors and obstacles at different levels, and it provides simplified feedback. The inertial sensor input was used to determine the orientation of the cane. To determine the tilt angle, (inclination), they used the gravity vector and its projection on the axes of the accelerometer. Therefore, the tilt’s angle of the device was measured using the *X* and *Y*-axis inclination of the accelerometer. Then, the inverse sine of the *X*-axis and inverse cosine of the *Y*-axis were processed to determine the inclination angle from the measured acceleration. The authors did not indicate which type of IMU they used. In [80], which is an expansion of previous work [87,88], the authors proposed the adaptive ground segmentation method for obstacle avoidance. For this method, they used the camera’s attitude angle measured by an IMU, and a corresponding 3D point cloud in the world coordinate system was created and merged with GPS data.

Finally, Fan et al. [66] developed a virtual cane based on an FPGA device (Xilinx ZYNQ-7000). This device is designed to build a map of obstacles in front of the user based on signals emitted by the devices: the laser light provided from the line laser is captured by a CMOS camera. Then, images with a line laser stripe were transferred to the FPGA device. Using the camera’s internal and external parameters, FPGA can calculate the true distance between obstacles and the camera. The user must swing the device horizontally so that the vertical light can scan all the objects ahead. Therefore, the IMU tracks the system’s pointing angle and relative position with respect to the world coordinate frame. Having both pieces of information, they calculate the distance and shape of obstacles in real time.

In this section, all the authors reported sensor fusion with at least one optical sensor, including also lasers and ultrasonic sensors to obtain information about the surroundings for obstacle detection and navigation [75,76]. The reported navigation error with the lowest value was 0.41 m, as shown in Table 3 by [73]. In this system, PDR is provided by the MPU-6050 and the sensor input includes GPS and an RGB-D camera. This system is the ARIANNA (Path Recognition for Indoor Assisted Navigation with Augmented Perception), which was focused on navigation without obstacle detection.

Other authors proposed the improvement of the functionality of the existing smart vision canes by adding functions such as an emergency call to send a GPS address if the person gets lost, or a remote-control feature to find the stick in case the person loses the stick. The system has an indoor and outdoor guiding system. In indoor systems, the acceleration and gyroscope input is used to count the steps and to verify the directions in which the steps are being produced, so the system can ensure that the person took the exact predefined path to the desired place by using the Adafruit wave kit for feedback to the user [81].

Li et al. proposed a framework to avoid objects in indoor environments [82]. This framework is composed of an RGB-D camera and IMU to detect objects and make a collision-free patch in real time. The acceleration and angular velocity of the IMU are used to obtain the real orientation and height of the camera that are necessary to create a ground segmentation. Decomposing gravity from three-axis accelerations allowed obtaining the camera initial orientation (pitch and roll angles). On the other hand, real-time orientation was calculated by integrating the gyroscope measurements. The initial height estimation was based on the distance between the chest camera position to the ground. A pedestrian crossing mobile application was developed for the blind by [83]. This system, which could send crossing requests to the signal controller via The National Transportation Communication for Intelligent Transportation System Protocol (NTCIP) without the need for pushing the conventional actuation push button, was a proposal for the traditional accessible pedestrian signal systems. In this system, the inertial measurement united from the smartphone was used to estimate the heading.

An augmented reality system was developed based on radar technology and internal sensors. In this system, measured distances get translated into an interpretable sound rendered in a virtual audio space. The relative orientation of the devices in the head and the hand are computed using the transformation matrix output of both IMUs. At the initial point, the startup of the system initializes the orientation. The azimuth and elevation angles were computed from the resultant matrix in addition to the radar sensor measures. The collected input was the database for the convolution processing [86].

Gill, Seth, and Scheme evaluated the effectiveness of a multi-sensor cane in detecting changes in gait proposed for the elderly and visually impaired; the IOT multi-sensor system included strain gauges to measure load. Different walking conditions, including impaired vision and walking abnormalities due to incorrect cane lengths of the volunteers, were tested by simulating walking abnormalities. The inertial measurement values were used to classify the walking cycle events [84].

A device capable of detecting and locating objects as an object manipulation aid was proposed by [85]. The hand-worn device was composed of an RGB-D camera and an inertial sensor that was used for pose estimation by Depth Enhanced Visual-Inertial Odometry (DVIO). The system provides electro tactile and audio feedback to the user.

### 3.4. Accelerometer, Gyroscope, and Magnetometer Fusion

Magnetometers complement accelerometers by providing sensor heading (orientation around the gravity vector), which is information that accelerometers alone cannot provide. With the fusion of accelerometers, gyroscopes, and magnetometers, the orientation is estimated based on the direction of the magnetic field. In addition, other embedded models that estimate pose can be obtained, which in many cases are more accurate models. In this section, the sensor fusion reported by the authors includes technology such as optical and ultrasonic sensors, BLE beacons, and GPS.

The complementation of the IMU sensor fusion with the mentioned sensors resulted in improvements in navigation system precision. The accuracy obtained with IMU sensors integrated in smartphones is less precise than that obtained with external IMU sensors. For instance, errors on the order of meters (from 1.5 to 6 m) were registered with internal smartphone sensors, while, with external sensors, errors decreased in the order of centimeters (6.17 to 104 cm). This is also attributed to sensor fusion (see Table 4).

**Figure 3:**
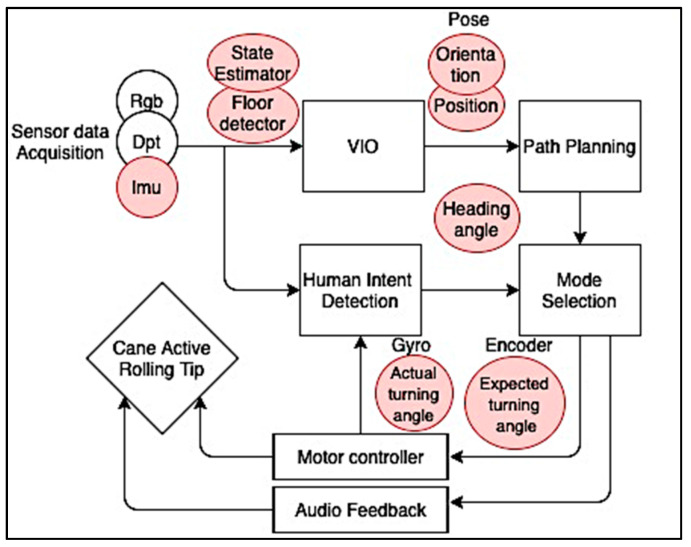
Adaptation diagram from the Robotic Navigation Aid system proposed by [89].

A Robotic Navigation Aid (RNA) system described by Zhang and Yen [89] used an RGB-Depth camera and inertial input from an VN-100 IMU/AHRS VectorNav IMU to acquire information about surroundings (Figure 3). In this prototype, the sensor fusion was used for the Visual Inertial Odometry (VIO), which estimates the RNA’s pose (orientation and position) so that the path planning node can use the position information to compute desired path and confirm the Desired Direction of Travel that would be used to control the active rolling tip. IMU measurements were used to calculate two of three components from the VIO: For floor detection, the gravity direction 𝑔⃗ and the inclination angle θ of RNA. The IMU state estimation calculates its own pose, velocity, and biases from its own measurements in conjunction with the extracted floor plane data, the tracked features, and the depth data. Then, these parameters are sent to the path planning node. The mode selection is performed automatically on the Human Intent Detection interface. The gyroscope indicates the actual turn angle in motion, which is compared to the expected turn angle from the encoder data (measuring the user’s compliance). If both turn angles are equal, this indicates to the system that it intends to use the RNA in its robocane mode instead of its with-cane mode (without robotic aid), expecting a motor-controlled motion of the tip.

The continuation of the previously developed work was also included in this review [85]. This article presented a new method to achieve more accuracy during the navigation. This method is mentioned above, DVIO, which integrates the geometric feature and the visual features of the scene with the inertial data for more acute estimation of the RNA’s pose.

Another navigation system was designed to aid VI mobility in places where there is no access to GPS, Bluetooth, or Wi-Fi [90]. The proposed method requires previously recorded paths using the inertial sensors of a smartphone for further guidance. The values obtained from the IMU were used to count steps and determine orientation to calculate route segments, distances, and turns. Vertical acceleration was used to estimate distance because it has a bigger amplitude than does horizontal acceleration. The average step length was calculated from a 20 m walk, and this information served as an input parameter to the program. The average azimuth of the segments was also calculated using the magnetic field perturbation from all three axes of the magnetometer compass. The system is similar to that of [91], who designed a smartphone-based indoor navigation application to aid the VI when using public transport. The system calculates pedestrian dead reckoning based on graphics, and it uses the existing tactile paths for positioning and navigation. Determining the attitude of the smartphone, relative to the user, was crucial to construct the pedestrian dead reckoning algorithm (PDR). The algorithm consisted of computing the orientation quaternion of the local-level frame relative to the body frame. The tilt components, such as roll and pitch (computed with acceleration data) and the heading component, were determined separately (computed with magnetometer data). The quaternion estimation involves a prediction step in which the angular rates (obtained from the gyroscope) are applied. With the step absolute orientation angle, if the step length is known, the change of position can be estimated; as the sensors from smartphones are generally low cost and not specifically designed for navigation, a PDR trajectory needs to be created by developing a map matching algorithm with a graph created with tactile paths (BLE beacons).

**Figure 4:**
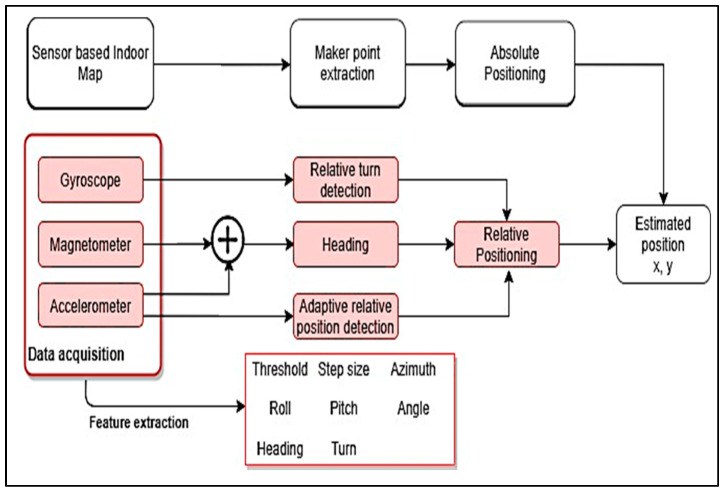
Framework of an indoor navigation system for VIP path by [30].

An indoor navigation system was developed based on the same sensor input as in [90] (inertial sensor from smartphone and Bluetooth beacons) [30]. A framework was proposed that combines relative position-based learning techniques from IMUs and absolute position based on iBeacons (Figure 4). In this framework, gyroscope data were used to detect relative turns and magnetometer axis fusion with accelerometer data provided heading detection. In addition, an accelerometer provided the adaptive relative position detection. With these three components, it is possible to obtain relative positioning, which, combined with absolute positioning, can provide a final estimated position. Features, including step size, orientation, standby position, roll pitch and yaw, relative turn, movement, and heading angle were all extracted from IMU sensors.

Considering the ability of a VIP to use auditory information to locate sound sources, a Real-Time Localization System (RTLS) for indoor and outdoor sports was designed by Ferrand et al. [92]. The system consists of an accurate 2D position system, a head tracker, and a sound renderer to simulate a virtual sound source. For this system, they used a BNO055 Bosch IMU sensor to determine the orientation of the body, in fusion with an Ultra-Wide Band sensor to provide precise distance measurement and an Optical Flow sensor to determine the velocity of the person when walking. They used the localization of the user to create an augmented reality scene with virtual sound. The software spatializes a sound depending on the position of the user, so the user could identify its own position in the track based on the sound that was being produced. In these systems, the Euler angles from IMU as the head tracker was essential for precision.

Ciabanou et al. [101] developed a system to detect indoor staircases with the help of an RGB-D camera. The algorithm is based on clustering patches from the normal map. To support information provided by the images, an IMU sensor was used to obtain absolute orientation to correct the normal orientation with respect to the depth sensor movement. No additional information about the characteristics of the input or orientation algorithm was provided by the authors, as there are several models to obtain absolute orientation and will be discussed in the next section. In the final algorithm, an important input, “T_angle_”, is referred to as a threshold for filtering by orientation. According to the authors, the mean rotation angle of every region was computed.

Simoes et al. [93] created an indoor wearable navigation system for users with visual impairments using computer vision, ultrasonic sensors, and an accelerometer, gyroscope, and magnetometer. The authors didn’t provide specific information related to the use of the inertial sensors but declared that during navigation, the path to the next marker was calculated based on the origin point or location point information, and on the orientation. When the system detects a marker, the user’s position, direction to other markers, arrival time, and others, are updated and enhanced. They improved the testing OpenCV algorithms to recognize static objects such as doors, walls, etc.

**Figure 5:**
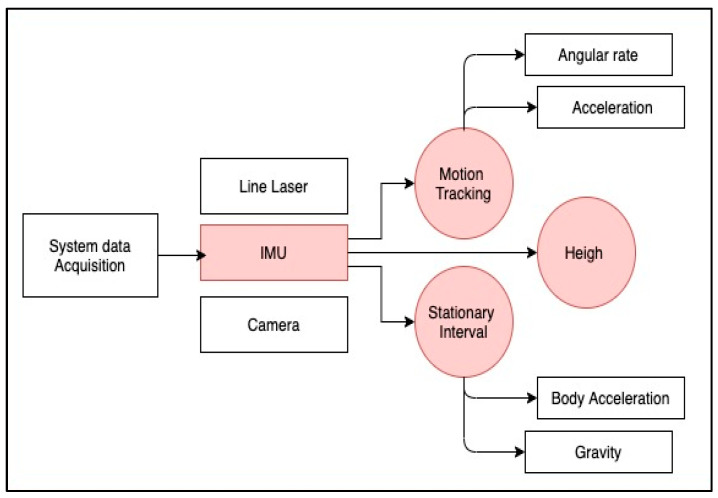
Adaptation diagram from the assistance system solution prototype and user’s swing motion by [94].

Dang et al. [94] created an assistance system in which the camera, IMU, and world coordinate frames were combined (Figure 5). Since the system combines multiple sensors, a calibration step was done first to estimate the relative position and orientation of each sensor. The height of the system was estimated based on the orientation of the IMU and the laser stripe distance. The motion of the hand was tracked using a Kalman filter. There is one moving interval and two stationary intervals with respect to the person’s body. The stationary interval of the IMU sensor was detected when gyroscope values for all three coordinates were approximately zero. The inertial measure was needed for the Kalman filter-based motion tracking algorithm, since the sensors provide the acceleration and angular rate of the system while moving. This algorithm uses the system’s initial orientation as the initial value to track the orientation of the system in each swing. Once the orientation of the system was tracked, it was used to determine the pointing angle of the system. For this, the pitch angle was estimated using gravity data. The height also was used to estimate the distance between the person and any obstacle detected.

Using a sensory substitution device (SSD) for VI, Botezatu et al. [95] proposed a 3D representation on the space conveyed by means of the hearing and tactile sensors. The IMU sensor (LPMS-CURS2) is used to track the head and body movement. The data acquired from the stereo and structure modules are synchronized with the data provided by the IMU sensor in order to make corrections in the stereo and depth frames. This fusion allows the system to identify the ground plane, doors, and other objects. The inertial measurement was essential to determine device orientation and track gravity orientation so that the camera frames are processed only when the device was parallel to the bottom lines for a correct estimation.

“blindBike” is an application that uses IMU sensor data to assist VIP who bike [96]. The “Road Following” module of this Android application uses 2D computer vision and statistical techniques to create a turn-by-turn route based on GPS map indications. Sensing in this application consists of a smartphone camera, location (GPS) services, microphone, audio output, accelerometer, gyroscope, and compass. The authors do not provide much information about how the inertial measurement units are used in this system. However, they do explain that sensing units are used to detect the right edge of the road and to direct the biker to maintain their route along the right edge as needed. The measured distance of the biker from the estimated right edge of the road determines if the user is on course or not; compass data are analyzed to determine if the bike’s heading conforms to what it should be.

**Figure 6:**
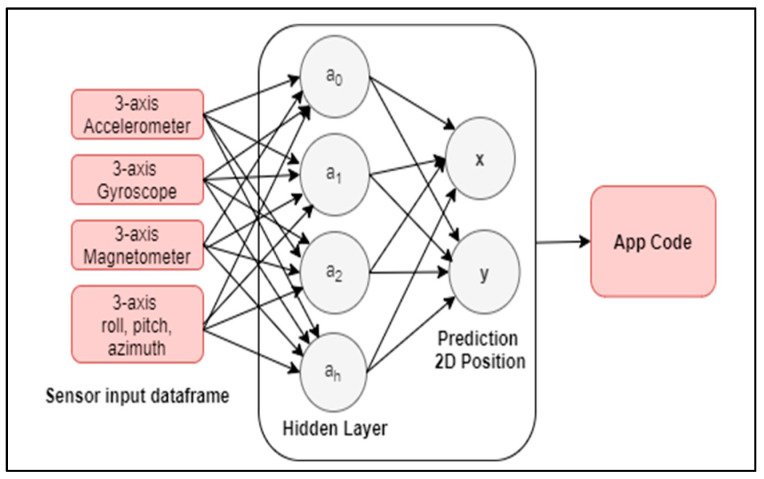
MPL network structure for the prediction of x and y position for indoor navigation by [99].

Mahida et al. [99] proposed to map the smartphone IMU measurements into 2D local coordinates using the regression-based training of Multi-Layer Perceptron (MLP). Within the three-axis values of the accelerometer, gyroscope, and magnetometer, plus the roll, pitch, and azimuth values from IMU, they trained the algorithm to predict the position of the VI user when holding the phone. They used a previous database that included two types of rooms dividing the spaces into microcells (x,y); the resultant output, predicts an x,y position for indoor location through an smartphone app, as shown in Figure 6.

Finally, a wearable low-cost system was developed by [97]. In this system, the authors calculate the magnitude of the acceleration, remove the gravity of the acceleration, and filter the resulting magnitude. The peaks of the magnitude that are above the standard deviation of one were counted as steps. The magnetometer is used to calculate the heading angle. Both are complemented with the obstacle detection system and provide real-time voice command instructions to the user to avoid obstacles. The physical obstacles and the people were constantly being detected by the Pi camera and ultrasonic sensors, respectively.

## 4. Discussion

### 4.1. Technical Analysis of the IMU’s Roles

#### 4.1.1. Motion Measurement, Angular Velocity, and Fall Detection

In the first section, most of the authors reported using ActiGraph wearable accelerometers to measure PA. These sensors have been used by the medical community for a long time as activity monitors in clinical trials such as analysis of health psychology and sleeping disorders [102,103], but most of them uses this sensor to measure physical activity [104]. The sensor provides raw three-axis acceleration with a sample ranging from 30 to 100 Hz, which is recorded and then processed. In the processing, these raw data can provide information about the motion activity, such as step counting and positional data (standing, sitting, or lying down). It has been proven that this type of accelerometer may not be sensitive enough to measure very low motion or low physical activity (which is sometimes the case of VIP), suggesting that for more accurate measurement, new classification of activity counts should be developed [105]. Motion detection by accelerometer readings, to establish if a person is moving or not in order to eradicate false vibration, was also applied by Trivedi et al. [59]. Motion readings can also be used for fall detection. Two different methods for fall detection excel in this revision due to the simplicity of the algorithms. Chen et al. [77] propose a threshold-based method where they calculate the acceleration vector sum (AVS) and the angular velocity sum (AVVS) to detect the movement of the user using the equations:

(1) $$\mathrm{AVS}=\sqrt{\left(a_{x}^{2}+a_{y}^{2}+a_{z}^{\;2}\right)}\;,\;\mathrm{AVSS}=\sqrt{\left(v_{x}^{\;2}+v_{y}^{2}+v_{z}^{2}\right)}$$

The data are collected consecutively while the system is active; it provides a fall alarm if both thresholds exceed the previously settled parameter, and a similar method is reported by Nkechinyere [67]. On the other hand, Ref. [78] used the orientation angles of a placed inertial sensor in order to measure the angle between the crutches (and canes) and the vertical of the world coordinate system, so that when a crutch or cane falls on the ground, an alarm is sent to the main developed system. Another simple method regarding angular velocity was reported by [72]. The sweeping velocity of the long cane was obtained using a gyroscope *Z*-axis signal (due to configuration of the placed sensor). They used this signal to calculate the frequency and speed of the sweeping. By establishing the quotient between the number of zero-crossings (determine a change of direction in the sweeping cycle) and the product of two times the diagonal duration in seconds, since a complete cycle was calculated from the initial position at the right most point and back from the left most point to the initial position of the subjects, in which the angular velocity is zero.

#### 4.1.2. Orientation/Attitude Estimation and Heading

The relative orientation of two devices can be estimated by computing the transformation matrix output of the IMUs placed in these devices. It starts by initializing the absolute orientation (orientation with respect to the world coordinate system). From this point, the orientation of one device is transformed into the device’s coordinate systems, which results in relative orientation. The azimuth and elevation angles can be computed from this matrix representation. Bai et al. [88] used the attitude angles to create an adaptative ground segmentation using a rotation matrix. For this goal, they compute a similar algorithm to that mentioned above, with the difference that the authors harness a depth image to create a 3D point cloud in the world coordinate system, where the reconstructed points x_w_, y_w_, and z_w_ are calculated by:

(2) $$x_{w},\;y_{w},\;z_{w}=z{\mathrm{EK}}^{-1}\;\left[\begin{array}{c} u\\ v\\ 1 \end{array}\right],\;\mathrm{where}\;E=\left\lceil \begin{array}{ccc} Cos\;\gamma & -Sin\;\gamma & 0\\ Sin\;\gamma & Cos\;\gamma & 0\\ 0 & 0 & 1 \end{array}\right\rceil \left\lceil \begin{array}{ccc} 1 & 0 & 0\\ 0 & Cos\;\alpha & -Sin\;\alpha \\ 0 & Sin\;\alpha & Cos\;\alpha \end{array}\right\rceil$$

The pixel value of point p(*u*,*v*) in the depth image represents the distance between the final point and the camera, which is equal to *z*. K is the camera intrinsic parameter matrix. The rotation angles of interest are the pitch α and roll γ angles corresponding to the *X* and *Z*-axis of the camera.

Absolute orientation can be directly obtained by sensor fusion in 9DOF sensors through Euler output angles [92,98] or quaternion estimation using inertial and magnetic observations, as in the case of [91], where the tilt or inclination components (roll and pitch) were determined separately from the heading component (yaw), so that there would be magnetic disturbances only in the heading value.

The gravity direction estimation has an important role in most of the developments that use inertial sensors for PDR or for absolute orientation. The direction of gravity can be considered as the unit vector perpendicular to the local horizontal in a typically northeast plane, pointing vertically downwards. This vertical direction vector is generally time-varying due to the rotation motion when expressed in the device’s coordinate system [106]. When the gravity direction is estimated in the device’s frame, it can be utilized to decompose any vector. In the case of [70], the gravity direction was used to determine if the device placed in the abdomen of the swimmer was orthogonal to the floor of the pool; in these intervals, the user’s position is estimated in frames captured by camera.

On the other hand, in the case of [82], the initial orientation of an object was calculated by decomposing gravity from three-axis accelerations, which was represented as:

(3) $$\mathrm{Pitch}=\tan^{-1}\left(\frac{\sqrt{A_{X,OUT}^{2}+A_{Y,OUT}^{2}}}{A_{Z,OUT}^{}}\right)\;\mathrm{Roll}=\tan^{-1}\left(\frac{A_{X,OUT}^{}}{\sqrt{A_{Y,OUT}^{2}+A_{Z,OUT}^{2}}}\right)$$

$A_{X,Y,Z\;OUT}$ are the accelerations along the *X*, *Y*, and *Z*-axis, subsequently. Since, in theory for real-time orientation, the estimation can be obtained by integrating the output of the gyroscope, as mentioned before, the estimation suffers from the integration of drift over time. The authors used a complementary filter to mitigate the noise and the horizontal acceleration dependency in real-time orientation.

The heading estimation (yaw angle) can be obtained either from inertial sensors only (accelerometer and gyroscope) or accelerometer fusion with magnetometer. In any method, a more precise measure of the roll and pitch angles is easier to obtain than a precise heading measure while calculating orientation angles, which is due to the magnetic disturbance when using a magnetometer or accumulated drift error when using gyroscope values [57,107,108]. These effects can be limited with magnetic perturbation compensation algorithms or with the domain of specific assumptions when treating the sources of error [109].

Although both methods are used according to the hardware selection of the authors, a more precise heading can be obtained when using magnetometer, since the orientation can be estimated based on the direction of the magnetic field [108]. In [97], this method is applied using the next equation for yaw (heading) estimation:

(4) $$\mathrm{Angle}=180\;x\;tan^{-1}\;(\frac{my}{mx})/\pi$$

where *my* and *mx* are the magnetometer reading of the *Y* and *X*-axis, respectively; to capture the magnetic energy around the surface of a sensor, mechanical calibration is needed.

#### 4.1.3. Positioning and Tracking

Pose estimation is referred to the estimation of both orientation and position by modeling the accelerometer and gyroscope measurements to the dynamics [108]. In dynamic models, for the estimation of states from multiple sensors, the most widely used technique is the Kalman filter, which uses an optimization method for estate estimation [110]. The difference of this method with the Extended Kalman filter is that the Extended method computes filtering estimates in terms of the conditional probability distribution, while the other method can be interpreted as Gauss–Newton optimization of the filtering, using normal distribution in the processed and measured noises of the sensors. The Kalman (KF) and Extended Kalman filtering (EKF) algorithms are used to compute pose and tracking on linear and non-linear models [57,108,110,111].

A Kalman filter-based algorithm was implemented by [76] using the angular rate and accelerations in order to estimate the pose of the system, with the assumption that an accelerometer measures only the gravity when three coordinates of gyroscope values are all near zero, since according to the authors, the acceleration of movement of the visually impaired is small during normal walking. With this presupposition, the pose of the system with respect to the temporary world coordinate frame can be calculated. The Kalman filter-based motion tracking algorithm was also applied by [94]. On the other hand, Croce et al. [73] implemented the Extended Kalman filter. For the state model, the position estimation is calculated on the IMU-based PDR algorithm, using only accelerometer and gyroscope measurements. The user heading, absolute velocity, and coordinates were considered in the measurement model.

This KF-based algorithms may not apply to all positioning estimation scenarios; some other non-linear models that are derivatives of the EKF are also suggested by authors to face the estimation accuracy problems of the sensor states [112]. However, the KF-based algorithm is the most frequent method used by the authors of the selected articles discussed in this review.

The positioning models can be improved with hardware implementation when fused with local markers as in the graph-based PDR model presented by [91] or the proximity based on visual pattern model by [93]. It can also be improved by using local markers and the implementation of deep learning models [30,99].

### 4.2. Usability

As mentioned in the introduction, an important factor constraining the development of systems to aid the VI is the acceptance of these technologies by the visually impaired community. The following section summarizes information from the research articles presented in this systematic review that pertains to the participation of VIP in tests of proposed systems.

Apart from the six articles focused on the measurement of PA, which are based on the participation of VI; only eleven of the 34 remaining articles summarized in the review tested proposed systems with VI volunteers or include VI during their experimental phase [64,67,72,73,79,80,91,92,93,95,98]. Three reported tests used blindfolded (BF) volunteers [59,89,97], and one used both VIP and BF [90]. There is elevated participation of VI in the Bai et al. [80] and Meshram et al. [79] papers. However, most of the authors did not include (or mention) usability questionnaires or user feedback after the usability testing; comments about the user-centered approach are shown in Table 5.

Concerning usability questionnaires after prototypes testing, only three of the 40 articles reported questionnaires of experience or qualitative feedback after VI participation in the tests, and two reported grading performance or obtaining qualitative feedback from BF participants. The relatively few questionnaires mentioned solicited feedback information such as ease of use or wearability usefulness; response time; independence and localization; sense of safety; and advice for future modifications [93]. Nor did they mention the extent to which the system was helpful and general usability [80].

The paucity of user feedback reported is alarming. VIP and volunteer feedback are important to the development of such systems; targeted users must be considered during the development process or usability may be compromised.

### 4.3. Field of Applications

**Figure 7:**
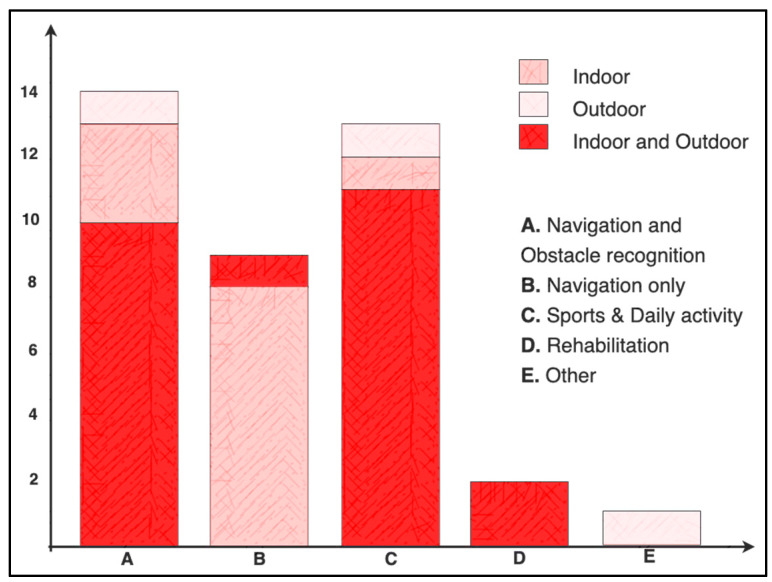
Distribution of the articles selected in the systematic review according to the fields of application.

“Navigation and Object recognition” was the most prevalent VIP application and was cited in 38% of systematic review articles (15 articles), as shown in Figure 7. A high percentage of articles (40%) in this application did not specify which kind of IMU was used as sensor input; this was likely because algorithms for identifying obstacles were based on camera sensing (67%) and/or ultrasonic or laser sensing (67%), so the authors provided more details about the specification of these sensors. However, IMU fusion sensors (accelerometers and gyroscopes or accelerometers, gyroscopes, and magnetometers) played essential roles in the articles selected in these field of application. The roles vary from motion detection and tracking to pose, attitude, or orientation estimation and step counting or fall detection (see Table 3 and Table 4).

“Navigation only” was the second most cited field of application and was represented in 23% of the reviewed articles. Researchers tended to use IMUs for several tracking purposes in the systems described: primarily PDR, pose estimation, and step detection. Smartphone IMUs were used as input to algorithms in more than half of the articles to provide “simple architecture” solutions. Only one article reported the use of a single IMU measurement (gyroscope) for input, while 78% developed devices or systems that used three measurement inputs: accelerometer, gyroscope, and magnetometer. The authors of one article did not declare the type of sensor input but referred only to “IMU” input. However, given the nature of the extracted data (absolute orientation), it can be assumed input was from at least two sensors. Although the IMUs listed (ADXL, MPU, LPMS-CURS2, and Xsense) are reported to be more accurate when compared to smartphone IMUs, one article reported the use of smartphone inertial sensing for “Navigation and Object Detection” as compared to five articles in the “Navigation only” application field.

About 33% of the articles were categorized as dealing with “Sports/daily activity” application. We included articles that described or reported research focused on measures of physical activity of VIP, monitoring or improving QoL activities, and systems developed to aid athletes with visual impairments. Research that focuses on monitoring physiological and behavioral patterns with wearable devices, such as inertial sensors, has become more prevalent due to the increasing availability of wearable small devices [113]. Wearable accelerometers are widely used in adapted PA research, since physical inactivity is a serious health issue in VIP [61]. Six articles regarding the measurement of PA among VIP are included, which represent 46% of the articles cited in this section. On the other hand, participation in sports is proven to benefit the VI, not only physically but also personally and socially [114]. The International Blind Sport Federation includes athletics, chess, goalball, soccer, judo, bowling, powerlifting, shooting, showdown, swimming, and Torball as sports. However, climbing, baseball, cricket, golf, sailing, and rowing are also practiced by VIP [115], but only four aid systems related to running, swimming, biking, and roller skating were found in the systematic review.

The IMUs in this application field section were used basically to measure PA by wrist-worn accelerometers to monitor daily life activity by using accelerometer sensor input only. For sports, the roles of the inertial sensors depend on the four identified sports: running (sense the foot movements), biking (detect the right edge of the road), swimming (track the direction of gravity), and roller skating (body orientation and head tracking). Fifteen percent of the articles in this section reported the use of smartphone IMU sensing; 46% reported the use of ActiGraph accelerometers. The Bosch BNO055 and BMI055, the KXR94-2050, and the TDK Inversense MPU-9250 were used in different systems in this application field. For input, 61% of the review articles in this application field used accelerometer data exclusively; one reported using a gyroscope, while four used sensor fusions of acceleration and gyroscope.

Rehabilitation is the systematic process in which VIP are provided with tools to help them deal with their visual impairments with greater independence and self-confidence. These tools include activities such as learning Braille, learning how to use a cane, sightless feeding of themselves, and optimizing the use of residual vision and teaching skills in order to improve visual functioning in daily life [116], as well as other daily activities such as orientation and mobility trained by specialists in rehabilitation [2,4,117]. Only two of the reviewed articles seem to have a rehabilitation approach, although many other designs included in the review can be used in rehabilitation [6,118,119]. However, advances in inertial sensor technology have been critical in assisting in the rehabilitation processes of other physical disabilities such as orthopedic [7,120,121,122,123,124,125]; no further developments have been found to have the specific approach of this important stage in the life of a visually impaired person, even though the importance of this stage has been proven [117,126]. Since the loss of vision leads to functional disabilities and restrains the participation in everyday activities, it limits the individual’s autonomy and QoL [127]. Only 13% of the selected articles featured systems to aid the visually impaired people when practicing sports; this is a fact that deserves attention, because as mentioned before, there is a need to promote PA with visual impairments, since inactivity is an alarming and common health issue along them. One last article was considered as “Other” according to the application field division; the authors proposed a system to aid pedestrian signals through a “Virtual Guide Dog” app. Sixty percent of the reviewed articles reported having an indoor and outdoor focus, which relates to the number of articles in the navigation and obstacle detection and the navigation-only applications, while 33% of the articles were focused on developments for indoor only and 8% were focused on outdoor only.

### 4.4. New Avenues of Research and Missing Elements

#### 4.4.1. Artificial Intelligence Integration

The integration of IA architectures was found in twelve of the reviewed assistive technologies; nevertheless, only 17% of these articles (2) used the IMU sensor as input (data feeder). Instead, the rest of the authors used optical sensors from camaras as an algorithm’s input. This is because most of the developments were focused on object detection, object recognition, and obstacle avoidance. In this case, several machine learning architectures were tested, such as decision tress [100] and class labeling on computer vision [93]. Deep learning, which consists of networks that have the particularity of extracting features automatically, were applied to the rest of the articles cited in this section, of which Convolutional Neural Networks (CNN) was the most tested architecture [73,77,82,87]. In this review, the authors that implement inertial sensors-based IA applied the architectures to obtain the prediction of a position in 2D local coordinates (x, y) [99] and for recognition of human activities [67]. In the case of the position prediction, an MLP neural network based on regression was tested, reaching an accuracy of 94.51%, which represented a 0.65 m positioning error by using accelerometer, gyroscope, and magnetometer input. This deep neural network model was suggested by the authors as a complementary system for the previous indoor navigation framework, which is also discussed in this review [30]. For human activity recognition, the authors validate a method of neural network regression, achieving 100% accuracy in activity classification using accelerometer input only. IMU data processed as time series is a method for preprocessing the raw data from inertial sensors that has emerged lately, and that helps improve the accuracy of the predictions on human activity recognition [128,129,130,131]. On the other hand, AI based in inertial sensing can be used to improve the parameter estimations in the geometric motion models; also, it can be used to replace the filtering complex models in the nonlinearity scenarios, for instance to estimate pose and tracking [132]. This would improve the accuracy problems in navigation, where most of the assistive devices of the review are focused. Having said that, it is important to mention that a new avenue of research is the artificial intelligence-based inertial sensing—for instance, for navigation applications.

#### 4.4.2. Biomechanical Analysis

The biomechanical research of visually impaired people is an important research field for injury prevention and for evidence-based rehabilitation methodology. Most of the medical research found in the review was focused on evaluating the physical activity of people with visual impairment; however, visual impairment is usually accompanied by age-related degenerative diseases. That is why a part of the literature focused on visual impairment and blindness is dedicated to study the biomechanical analysis, such as parameters of gait and posture [133,134,135,136].

Analysis of gait parameters using inertial sensors, on the other hand, has been an important topic found in the literature for more than a decade [137]. This literature is focused on the development of algorithms to calculate the stride length and gait velocity as well as analyzing the gait cycles to identify abnormalities, diseases, or changes over time [138,139,140,141] for different medical applications. However, no studies in this review were found linking this technical study area to visual impairment. In fact, in the general literature, a research paper was found featuring a dataset of inertial sensor time series collected from blind walkers created by Manducci & Flores [130]. The authors provided these data to be used by researchers who are interested in personal mobility, since as the authors claimed, there are peculiar characteristics in visually impaired gait. For what we considered this topic, it is a missing element in the literature.

In addition, biomechanics and quantification of the long cane for analysis of the motion parameters and performance using inertial sensors is also a research field that can be considered as a new avenue of research and a missing element. Since as shown in the few studies that are found in the literature regarding this topic, a sophisticated 3D motion analysis equipment has been required in both studies to conduct the motion acquisitions for further analysis [72,142]. However, due to the advances in sensor fusion, by using inertial sensors instead of the 3D motion systems and with the right pre-processing and proper interpretation, motion analysis and quantification of the long cane characteristics can be obtained.

### 4.5. Limitations

A limitation to the present review may be the fact that only three databases were included in the systematic article search. We selected three databases that we consider are relevant to the research topic; perhaps more articles addressing the use of inertial sensors in assistive technologies for visually impaired people are found in the literature but did not comply with the eligibility criteria and thus are not included because they are indexed in other databases.

Another limitation lies in the fact that there is no qualitative assessment of the selected articles; instead, all the articles found in the review that met the criteria were included for in-depth analysis, including conferences and short papers. As a result, this review provides a comprehensive systematic review of the recent literature, focusing on the discussion of the use of the inertial sensors.

## 5. Conclusions

A systematic review of research articles was conducted to find system developments that used inertial measurement unit sensors to aid the visually impaired people in assistive technologies. Reviewed articles were categorized according to the type of IMU input used and the role of these IMUs, including pose estimation (position and orientation of a body, an object of a white cane), identification of human motion, i.e., PA and falling, but also specific roles for each application field such as measurement of the sweeping velocity of a white cane, detection of the right edge of the road for blind bikers, or tracking the direction of gravity when swimming. The major approach of the findings was sensor fusion of accelerometers, gyroscopes, and magnetometers (35%), while the less common approach of the findings was the use of gyroscope input (10%). In addition to the IMU data, sensor fusion in most of the articles included GPS (20%) and optical sensors such as RGB-D (16%) and RGB (15%) cameras as ultrasonic sensors (16%). Second, better precision in navigation and positioning estimation can be achieved when there is a fusion of UWB, line lasers, and velocity sensors in hardware when implementing local markers and deep learning architecture in software development. The smaller errors in navigation reported were due to IMU sensor fusion with an RGB camera sensor and the use of external inertial sensors, for instance, MPU-6050 and VN-100 IMU/AHRS instead of a smartphone inertial sensor, which is less precise. In many of the articles summarized, “simple” architecture of the systems to aid the athletes is observed, suggesting that the use of inertial sensors is quite applicable in this area, only more knowledge of the specific activity is needed to create an assistive system. In addition, by using accelerometers only, ActiGraph IMUs were the most commonly used, due to the function of measure of PA. The results indicate that there are new avenues of research within the integration of AI with the use of inertial sensors as feeders to improve the accuracy of the assistive devices developed as navigation assistance. There are also missing elements in the literature such as technological developments to aid the rehabilitation process, the use of inertial sensors for biomechanical analysis in gait and posture parameters, and also biomechanics of usage of the long cane within VIP. In addition, the results have shown that it is necessary to promote as well the inclusion of technology in these biomedical research areas. Finally, a significant limitation evidenced by this review is the fact that the designed aids for the visually impaired lack user-centered designs. Most of the authors used blindfolded persons instead of actual blind persons during the validation of the developments, and only 8% of the reviewed developments included a usability questionnaire for the VI, which should be considered for future research.

## Figures and Tables

**Table 1:** Summary of reviewed articles in the accelerometer section.

Role	IMU	Sensor Fusion	Ref.
Identify human movement	ADXL345	RGB Camera, Ultrasonic Sensor	[59]
Measures physical activity	ActiGraph GT3x	N/A	[60]
Measures physical activity	ActiGraph wGT3X-BT	N/A	[61]
Measures physical activity	ActiGraph wGT3X-BT	N/A	[62]
Measures physical activity	ActiGraph wGT3x+	N/A	[63]
Sense the foot movements	KXR94-2050	N/A	[64]
Measures physical activity	ActiGraph GT3x	N/A	[65]
Measures physical activity	ActiGraph GT3x	N/A	[66]
Monitoring of human activities	Not specified	N/A	[67]

**Table 2:** Summary of reviewed articles in the gyroscope section.

Role	IMU	Sensor Fusion	RE	Ref.
Measure the tilt angle	MPU-6050	Ultrasonic sensor, GPS	0.2–0.5 FA/m	[69]
Detect rotation and movements	MPU 6050	Ultrasonic sensor, GPS	1–7 cm	[70]
Track the direction of gravity, orientation	Smartphone IMU	Camera	-	[71]
Sweeping velocity	Re Sense	Camera	-	[72]

RE = Reported Errors.

**Table 4:** Summary reviewed articles in the accelerometer, gyroscope, and magnetometer fusion section.

Role	IMU	Sensor Fusion	RE	Ref.
Device and pose estimation	VN-100 IMU/AHRS	RGB-D camera	0.2 m	[89]
Step counting, body orientation	Smartphone IMU	Barometer	-	[90]
Attitude estimation and orientation	Smartphone IMU	Beacons, GPS	5–6 m	[91]
Pedestrian dead reckoning	Smartphone IMU	Beacons	1.5–2 m	[30]
Body orientation	BNO-055	Optical flow sensors, UWB	0.5 m	[92]
Path calculation	Not specified	RGB camera, ultrasonic sensor	-	[93]
Acceleration and angular rate	Xsens IMU	RGB camera, line laser	6.17 cm	[94]
Tracking the head and body movement	LPMS-CURS2	RGB camera, structure sensor PS1080	25–104 cm	[95]
Detect the right edge of the road	Smartphone IMU	RGB-D camera, GPS	-	[96]
Step counting and Heading estimation	MPU-9250	Ultrasonic sensor, Pi Camera	-	[97]
Head tracking	MPU-9250	GPS/GLONASS and UWB	10–20 cm	[98]
Positioning and step size estimation	Smartphone IMU	N/A	-	[99]
Pose estimation	VN 100 IMU/AHRS	RGB-D Camera	1.5 m	[100]
Absolute orientation	Smartphone IMU	RGB-D Camera	-	[101]

**Table 5:** Summary of the visually impaired participation and usability discussion.

Visually Impaired Subjects	Usability Test	Usability Questionnaire	Commentary	Ref.
7	Yes	No	Subjects were participants on the blind marathon sponsored by Japan Blind Marathon Association.	[64]
1	No	No	-	[67]
10	N/A	No	Subjects recruited through the foundation Access for All (Swiss nonprofit organization).	[72]
No described	Yes	No	-	[73]
60	Yes	No	There were 30 subjects who were totally blind and the others had low vision. In addition, the authors involved physiotherapists, rehabilitation workers, and social workers for the development of the usability test.	[79]
20	Yes	Yes	Ten subjects were totally blind and the others were partially sighted. The authors followed the protocol approved by the Beijing Fangshan District Disabled Persons’ Federation for recruitment and experiments.	[80]
3	Yes	No	The subjects were student volunteers from the university. A mobility and orientation instructor evaluated their traveling techniques with a long cane to use the application.	[90]
11	Yes	No	The system was implemented and tested at the railway station in Graz, Austria.	[91]
2	Yes	No	-	[92]
10	Yes	Yes	The navigation profile of the users was considerate (height, walking speed, and step distance).	[93]
No described	Yes	Yes	-	[95]
2	Yes	No	-	[98]
